# Blunted emotion judgments of body movements in Parkinson’s disease

**DOI:** 10.1038/s41598-021-97788-1

**Published:** 2021-09-17

**Authors:** Emmanuelle Bellot, Antoine Garnier-Crussard, Elodie Pongan, Floriane Delphin-Combe, Marie-Hélène Coste, Claire Gentil, Isabelle Rouch, Marie-Anne Hénaff, Christina Schmitz, Barbara Tillmann, Pierre Krolak-Salmon

**Affiliations:** 1 UMR 5292, Lyon Neuroscience Research Center (CRNL), CNRS, INSERM, U1028, Université Claude Bernard Lyon 1, Université of Lyon, Lyon, France; 2 University of Lyon, Lyon, France; 3 Clinical and Research Memory Center of Lyon, Lyon Institute for Elderly, Charpennes Hospital, Hospices Civils de Lyon, 27 rue Gabriel Péri, 69100 Villeurbanne, France; 4 Neurology Unit, Clinical and Research Memory Center, University Hospital of Saint-Etienne, 42055 Saint-Étienne, France; 5 Bordeaux Population Health Center, INSERM, U1219, University of Bordeaux, Bordeaux, France

**Keywords:** Cognitive neuroscience, Emotion, Neurological disorders, Neuroscience, Psychology, Neurology

## Abstract

Some of the behavioral disorders observed in Parkinson’s disease (PD) may be related to an altered processing of social messages, including emotional expressions. Emotions conveyed by whole body movements may be difficult to generate and be detected by PD patients. The aim of the present study was to compare valence judgments of emotional whole body expressions in individuals with PD and in healthy controls matched for age, gender and education. Twenty-eight participants (13 PD patients and 15 healthy matched control participants) were asked to rate the emotional valence of short movies depicting emotional interactions between two human characters presented with the “Point Light Displays” technique. To ensure understanding of the perceived scene, participants were asked to briefly describe each of the evaluated movies. Patients’ emotional valence evaluations were less intense than those of controls for both positive (*p* < 0.001) and negative (*p* < 0.001) emotional expressions, even though patients were able to correctly describe the depicted scene. Our results extend the previously observed impaired processing of emotional facial expressions to impaired processing of emotions expressed by body language. This study may support the hypothesis that PD affects the embodied simulation of emotional expression and the potentially involved mirror neuron system.

## Introduction

Parkinson’s disease (PD) appears to be a model of neurodegenerative motor disease, also inducing cognitive and perceptual disorders^1–3^. Numerous studies have provided evidence that the classic motor symptoms of PD (akinesia, rigidity and tremor) induced by the dopaminergic neuronal loss in the substantia nigra pars compacta^4^ are accompanied by a progressive pattern of cognitive impairments and abnormal emotional and social behaviors^1–3^.

The recognition of emotional states in others is one of the critical components of social behavior^5^. Emotional disorders in PD appear early in the disease course, involving all components of emotion^6^. They seem to be cross-modal, i.e., affecting the recognition of emotions from both voices and faces^6^. The impaired perception, assessment and recognition of facial emotional expressions observed in PD^3,7^ are associated with disease severity (even though also observable at early stages), visuospatial abilities, cognitive impairment, mood disorders and apathy^3,8^. Some behavioral changes observed in PD, notably behavioral dysexecutive disorders, could be related to an altered processing of social messages^9^. Emotion recognition deficit in PD is associated with neural changes in several areas, including basal ganglia, limbic (notably amygdala), paralimbic and neocortical associative areas, as well as with impaired dopamine transmission in the mesocorticolimbic pathways^3,6,10^. They may involve the mirror neuron system (MNS), according to the embodied simulation theory^3,11–13^.

Body movements are a powerful vector of social and emotional information^14^. PD patients are affected by motor symptoms and body movements limitations^4^, as well as impaired perception of movements^15^. A study investigated PD’s perception of biological motion, i.e. movements coming from actions of a biological organism, using videos of point-light walkers (with scrambled versions as foils), and revealed a deficit of PD patients in detecting human walking^15^. However, no study has yet assessed the processing of emotions conveyed by whole body movements. Whole-body motor disorders, such as hypokinesia, disrupt PD patients’ ability to use their own body language^16^. This disruption may affect the access to own motor representations and impair “motor resonance” processing underpinned by the hypothesized MNS, which is used to understand and recognize emotions in others^17^.

Our aim was to assess PD patients’ abilities to process emotions conveyed by body movements through a valence judgment task using “Point Light Displays” (PLD)^18^, allowing us to focus on mechanisms used to extract emotional information from motion per se, without the influence of other visual information of the body (e.g., muscles, clothes) and the face.

## Results

PD patients and healthy control (HC) participants did not differ in terms of age, sex, education and their scores of the Geriatric Depression Scale (GDS) (Table 1). The PD participants performed in the normal range for global cognition (Mattis Dementia Rating Scale = 136 ± 2.94) and visuospatial abilities (VOSP unfinished letters = 18.77 ± 1.09, location of figures 8.69 ± 0.85). The levodopa equivalent daily dose was 600 ± 268 mg (4 missing data). The average disease duration was 8.4 ± 5.5 years.

**Table 1:** Demographic and clinical characteristics of the participants.

	PD group	HC group	*p*-value
N	13	15	
Age (years)	70.54 ± 8.09	68.60 ± 5.46	0.59
Gender ratio (F/M)	8/5	9/6	1.00
Education (max. 3)	1.62 ± 0.96	1.33 ± 0.62	0.58
GDS	8.00 ± 4.16	5.87 ± 3.89	0.13

Mean ± SD are reported in the table. Statistical analyses were performed using Wilcoxon Mann–Whitney tests [for age, education and GDS score] and Fisher tests [for gender].

*GDS* geriatric depression scale, *PD* Parkinson’s disease patients, *HC* Healthy controls.

**Figure 1:**
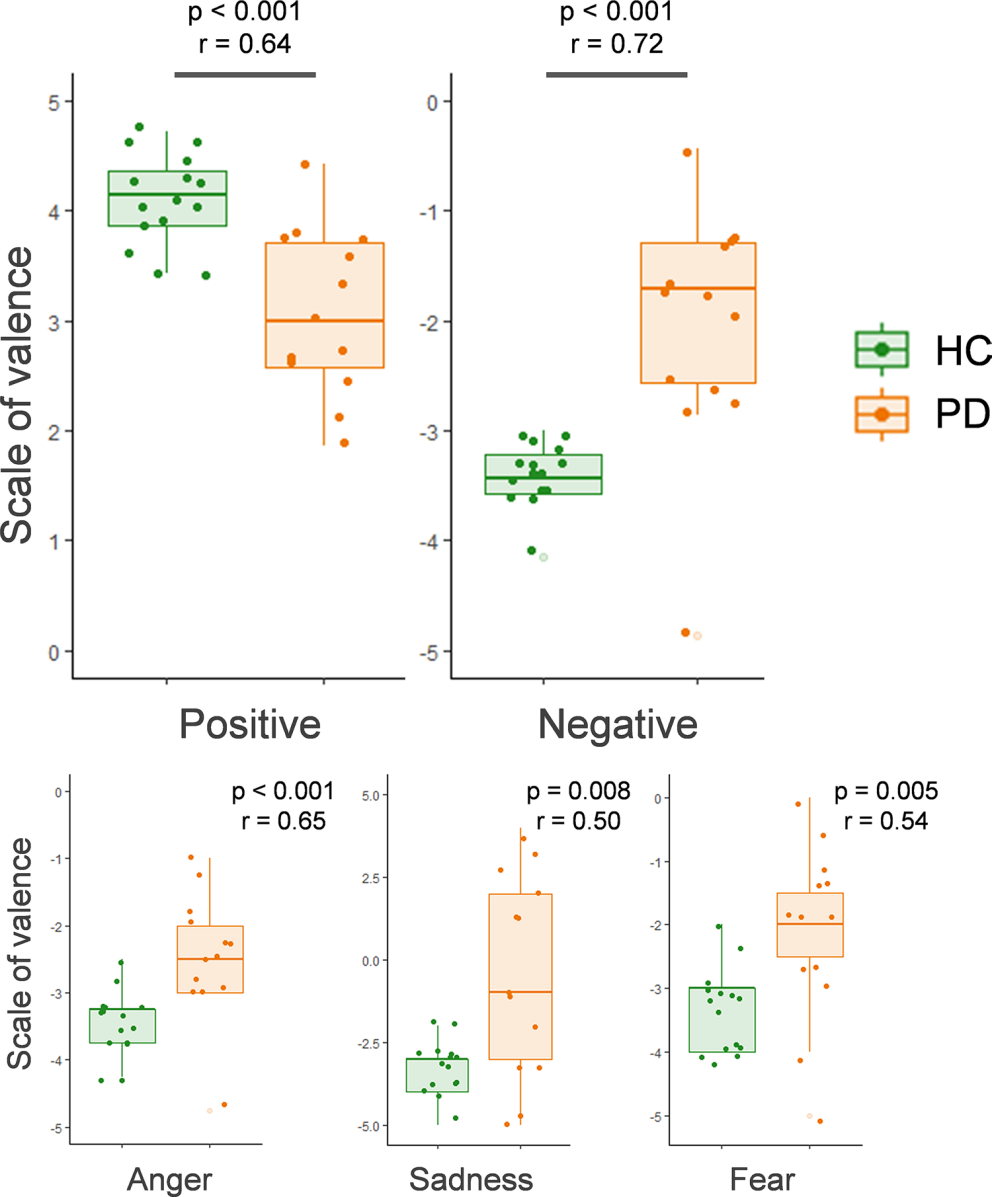
Boxplots comparing valence judgments of PD and HC participants, presented respectively as a function of the two main valence conditions (positive/negative) (top) and for each negative emotion separately (bottom). Note that the used subjective scale went from − 5 (very negative) to 5 (very positive). The y axis limits are [0;5] for positive emotions and [− 5;0] for negative emotions, except for sadness (y axis [− 5;5]). *P*-values (*p*) and effect size (*r*) of the Wilcoxon Mann–Whitney tests are indicated in the plot. PD = Parkinson’s disease patients, HC = Healthy controls.

The valence judgments of the emotional scenes were compared between participant groups for each valence (i.e. positive and negative) as well as, for completion, for each negative emotion separately (Fig. 1). For scenes with positive valence, PD patients’ ratings were significantly lower (i.e., less intense) than those of the HC group (respectively 3.08 ± 0.76 and 4.10 ± 0.41, *p* < 0.001, effect size *r* = 0.64, power = 0.98). For scenes with negative valence, PD patients’ ratings were also significantly less intense than those of the HC group (respectively, − 2.08 ± 1.09 and − 3.39 ± 0.29, *p* < 0.001, effect size *r* = 0.72, power = 0.98) (Fig. 1). A similar result pattern was obtained when analyzing each negative emotion separately (anger: PD patients − 2.46 ± 0.94 vs. HC − 3.43 ± 0.48, *p* < 0.001, *r* = 0.65, power = 0.90; sadness: PD patients − 0.46 ± 3.04 vs. HC − 3.33 ± 0.82, *p* = 0.008, *r* = 0.50, power = 0.89; fear: PD patients − 2.12 ± 1.36 vs. HC − 3.33 ± 0.65, *p* = 0.005, *r* = 0.54, power = 0.80) (Fig. 1). Results remain significant after Bonferroni correction for multiple tests. Results were overall similar when the incorrectly interpreted items (based on the results of the description task) were removed.

For the control condition, statistical analyses were performed on the average number of errors (= defined as an erroneous description of the scene; maximum number of errors = 14). There was no difference between errors committed by PD patients (1.0 ± 1.15) and HC participants (HC 0.80 ± 0.68), *p* = 0.92.

The clinical characteristics of the PD patients (i.e., cognition, disease duration and levodopa equivalent daily dose) did not correlate with measurements obtained in the task (i.e., emotion valence judgments), except for one case: global cognitive performance (evaluated with Mattis Dementia Rating Scale) correlated with positive emotions judgments (*r* = 0.69; *p* = 0.03, df = 11), but not significantly after Bonferroni correction for multiple test (Supplementary Table S1).

## Discussion

The aim of our study was to evaluate PD patients’ ability to judge the valence of emotional scenes depicted by body movements. Using a PLD material^14,18^ allowed for the isolation of emotional components conveyed by whole-body movements without facial expressions and other detailed information. While PD patients were cognitively unimpaired (as assessed by the neuropsychological evaluation) and were able to correctly describe the displayed visual scenes, they were impaired in their emotional valence ratings of these scenes, compared to healthy controls. Because of the same description accuracy in the two groups, PD patients’ impairment appears to be preferentially linked to an altered judgment of the intensity of emotion rather than to an alteration of the scene comprehension. This alteration appeared as a blunted rating of both positive and negative emotions, namely happiness, anger, sadness and fear.

Previous research using other materials, notably voice and faces, have reported PD patients’ deficit in valence judgments for emotional expressions, which is particularly acute for negative emotions (for example, fear, disgust, anger or sadness), but also exists for position emotions^6,19^. In line with these previous results, the effect size for differences between PD patients and HC were stronger for negative emotions than for positive emotions, albeit the differences were significant for both negative and positive emotions. The deficit in valence judgments for emotional expressions in faces has been linked to the depletion of dopamine in brain structures relevant for social cognition, such as the amygdala, orbitofrontal cortex and basal ganglia^6,20–23^. These structures play an important role in the processing and recognition of negative emotions as well as of positive emotions and high arousal level stimuli^24,25^. The possible alteration of supplementary motor area in PD^26^, an area involved in the processing of positive emotions^27^, may be partly responsible for the difficulties encountered by PD patients in judging the emotion of joy expressed by the body movements of others. The altered judgments of emotional movements in PD patients in our study suggest that the role of these brain structures extends to the processing of emotional scenes conveyed by whole-body movements.

Our study assessed PD patients’ judgments of emotional scenes depicting whole-body movements. Using visually reduced emotional body movements (implemented with the technique of PLD) seems particularly relevant to explore the MNS hypothesis. Indeed, PD is a model of motor production and motion detection impairment^4,15,28^. The ability to judge emotional scenes is partly enabled by simulation and thus direct understanding of actions/emotions expressed by the motor actions of others^12,29^. The integrity of the sensory-motor system seems thus critical for the recognition of others’ emotions^12^. The MNS has been shown to be involved in both the generation and recognition of facial expressions^11^. The embodied simulation theory suggests that “emotion recognition is facilitated by internally generated somatosensory representations triggered by the simulation of a perceived facial expression which activates the corresponding emotional state in the observer”^3,12^. Impaired motor dysfunction can lead to impaired emotion recognition^3,12,13^. This simulation of the emotional state of the executors perceived through their body movements allows the viewer to experience the feelings of the executors and has been shown to involve the activation of the MNS^29^. The MNS has been linked to numerous behaviors and abilities^30^, including (conscious or unconscious) imitation as well as emotion expression and recognition, which play a role in the communication of affective states^31^ and empathy^32,33^. Embodied simulation, an experience-based mechanism, is not the only functional mechanism implicated in emotion recognition. Emotions could also be analyzed and understood using explicit cognitive mechanisms, e.g. interpretation of visual aspects of the emotional scene^12^. These mechanisms are not mutually exclusive. In the present study, the reduced visual information in the stimuli (PLD) and their rapid presentation made less probable the use of perceptual strategies to overcome potential emotional difficulties, in particular, strategies applicable for face perception (i.e., the detection of infra-orbital triangle, nasolabial wrinkle raising and the involvement of zygomatic)^34^, and may argue for the embodied simulation mechanisms.

All patients in the present study performed the task during an “ON-state”, *i.e.* one hour after having taken medication, suggesting that treatments are not able to fully restore the ability to recognize emotions conveyed by body movements. Some previous data have suggested that dopamine substitution can modify biological motion perception in PD^35^, and further research is needed, in particular to investigate patients during an “OFF-state” with our present paradigm. In PD, dopaminergic depletion and neural degeneration underlie motor symptoms and may disrupt motor resonance mechanisms in limbic structures, like the ventral striatum, anterior insula and amygdala^20^. These structures play an important role in the empathic process^6,29,32^. Their degeneration in PD may alter this mechanism and consequently alter the recognition of emotion of others^6,10^. Our results could be partly explained by the dysfunction of PD patients’ “motor resonance”. This is consistent with a study reporting a decrease in PD patients’ spontaneous facial activity in response to emotional video clips^36^. This spontaneous imitation has been shown to facilitate individual empathic abilities^32^, and their diminution may also lead to alter the valence rating of emotions, in our present case conveyed by whole-body movements. Furthermore, it is important to note that our findings cannot be explained by potential depression in our patient population. Indeed, as previous research has shown impaired recognition of emotional facial expressions in depressive older population, our study had excluded participants with depressive symptoms^37,38^.

Although reasonable power for analysis was reached, the present study remains limited by the small size of the subject samples. Therefore, our results need to be replicated on a larger population. Another limitation of our study is the absence of a more fine-grained control task and a neutral condition. Even if we ensured the participants’ understanding of the scene by asking them to give a brief verbal description of the perceived scene, the description of the content of the scene does not enable to control for the capacity to process or detect rhythmic complex features. The main strength of this study is the originality of the task, with short movies depicting emotional interactions between two human characters presented with the PLD technique. The valence rating task did not require the production of semantic labels, as needed in previously used categorization/labeling tasks^19^, and, as previously mentioned, the reduced visual information in the stimuli and the rapid presentation limit the use of perceptual strategies.

Future research is needed to replicate these results with a larger patient group. Moreover, the influence of the medication on the processing of emotional whole-body expressions in PD and by this way on the MNS functioning should further be explored, *e.g.* in assessing the emotion recognition in “OFF-state” and “ON-state” of PD patients. Neuroimaging approaches will further allow investigating the links between performance and brain atrophy or metabolic changes. If the dysfunction of the MNS in PD patients is confirmed to participate to the difficulties encountered to process the emotions expressed by whole-body movements, “mirror” therapy or “action-observation” protocol may represent rehabilitation strategies in PD^39–41^. Such protocols may be beneficial to help patients with motor and non-motor symptoms, which impact on social interactions.

Our study examines PD patients’ ability to judge emotional scenes conveyed by whole-body movements using the dynamic technique of “Point Light Displays”. The blunted valence ratings for positive and negative emotions observed in PD patients may be the expression of a dysfunction of the embodied simulation process underpinned by the MNS. This impairment may be linked to PD patients’ motor disorders altering the mechanism of motor resonance and therefore the ability to understand emotional body movements.

## Methods

### Participants

Thirteen patients with idiopathic PD recruited at the Charpennes Hospital (Hospices Civils de Lyon, France) participated in this study. All patients were under dopaminergic therapy (levodopa or dopaminergic agonist medication). Exclusion criteria were, major cognitive deficits with scores inferior to 129 across the Mattis Dementia Rating Scale^42^ and visuospatial or visuo-perceptual disorders.

Fifteen healthy controls who reported no history of neurological or psychiatric diseases were recruited through various associations in the French region of Lorraine. A screening battery showed the absence of significant cognitive deficits in the control group: Mini-Mental State Examination (MMSE)^43^ (scores for all participants were superior to the exclusion score of 24), the 5-words test^44^ (all scores superior to the exclusion score of 10) and the clock drawing task^45^ (all scores superior to the exclusion score of 12).

The two participant groups were matched with respect to gender, age and education and gave written informed consent to participate in this study. They did not display any significant difference with regard to mood, as measured by the GDS^46^ (See Table 1 for details of participants’ characteristics). All participants were native French speakers. All methods were carried out in accordance with relevant guidelines and regulations. The study protocol, inclusion and consent procedure were reviewed and approved by a national ethics committee (*Comité de Protection des Personnes*, South-East III).

### Stimuli

Fourteen 3-s silent movies depicting whole-body movements presented with PLD were selected among the stimuli of Centelles et al.^14^. These movies showed emotionally loaded interactions between two actors, each of them being depicted by 20 white dots moving on a black background (e.g., for happiness: “two people dancing together”). The stimuli were selected on the basis of two pre-tests with younger participants (n = 14) as well as older control participants age-matched to our patient group (n = 5). In these pretests, each movie was judged regarding its valence (using a subjective scale from − 5 to + 5), its emotion and completed by an oral description of the perceived scene. They were presented on a 15-inch computer screen of a MacBook (OS X, processor 2.4 GHz). Among the 14 selected videos, 7 had negative valence (1 depicting sadness, 3 depicting fear and 3 depicting anger) and 7 had positive valence (happiness). PsyScope software^47^ was used for stimulus presentation and data collection.

### Procedure

Because of PD patients’ motor impairment, the experimentation was done at patients’ home. To maintain the same conditions for the control group, HC participants were also tested at home. PD patients were tested during the medication effect (*i.e.* during the “ON-state”, one hour after having taken medication). The total duration of neuropsychological pre-tests and the main experiment did not exceed 2 h, the patient thus remaining under the medication effect.

**Figure 2:**
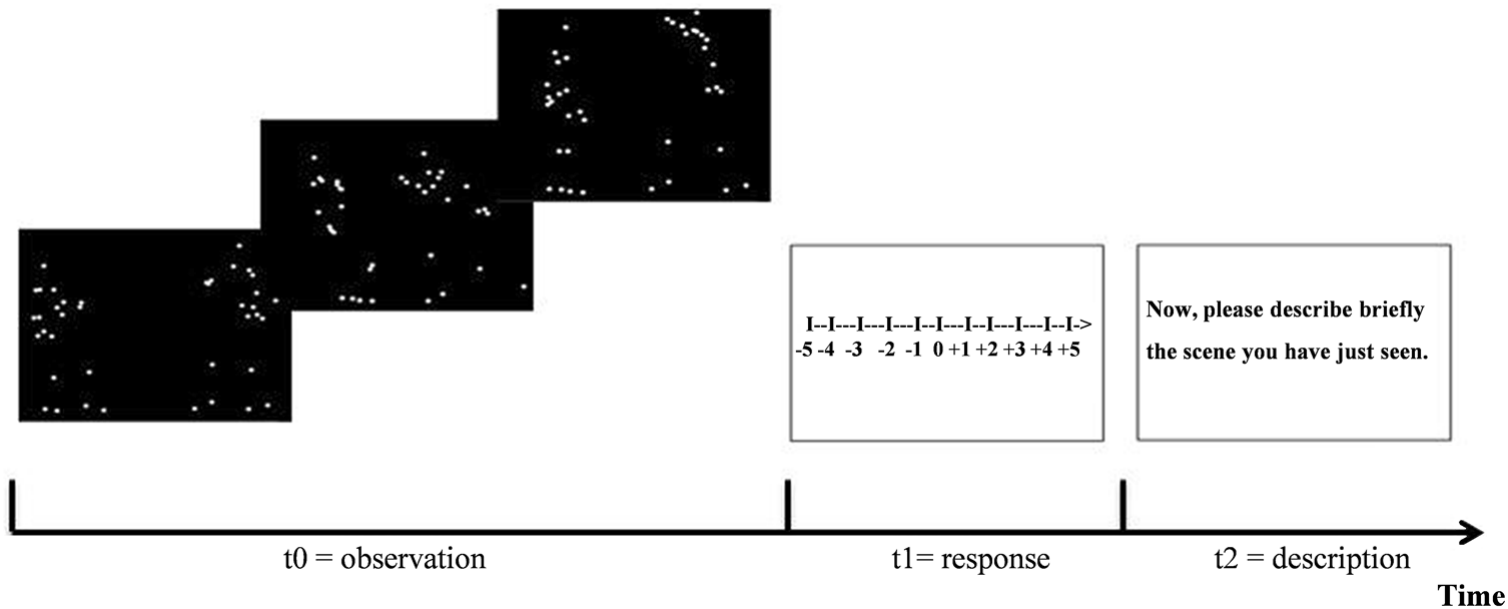
Experimental paradigm: valence judgments of emotional body movements, using the technique “Point-Light displays”. t0 = 3-s silent movies depicting whole-body movements presented with PLD; t1 = valence judgment task; t2 = description task.

The task was explained with two examples (one with positive emotion and one with negative emotion). For each trial, the movie was presented twice consecutively. Participants were then asked to judge the emotional valence of the scene by rating it as accurately as possible on an 11-point scale from − 5 (very negative) to 5 (very positive). Participants’ responses were then entered by the experimenter on the computer keyboard (Fig. 2). Participants were asked to give a brief verbal description of the perceived scene to ensure their understanding of the scene (control task). Participants’ descriptions were also entered by the experimenter via the computer keyboard (Fig. 2). The movies were presented in different random orders for each participant.

### Statistical analyses

Statistical analyses were performed using the R software version 3.5.2, 2018 (R Core Team, www.R-project.org). Values are reported as mean ± standard deviation (SD). We compared the sociodemographic and clinical variables between the two groups using nonparametric Wilcoxon Mann–Whitney tests for continuous variables and Fisher tests for categorical data. For the comparison of valence judgments between the participant groups, Wilcoxon Mann–Whitney tests were performed, effect sizes r were calculated as Z statistics divided by the square root of the sample size, and power analyses were realized using G*Power^48,49^. Correlations in PD patients between emotions valences and clinical characteristics (cognition, disease duration, levodopa equivalent daily dose) were performed using Pearson correlation tests. Bonferroni correction was conducted for multiple test correction. The alpha level for all tests was set to 0.05.

## Supplementary Information

Supplementary Information.

## Data Availability

The datasets used and/or analyzed during the current study are available from the corresponding author on reasonable request.
