# Supplementary material for: Blunted emotion judgments of body movements in Parkinson’s disease
Source: Sci Rep. 2021 Sep 17;11:18575. doi: 10.1038/s41598-021-97788-1 (PMC8448734; doi:10.1038/s41598-021-97788-1)
Supplement: Supplementary file 1 — Supplementary Information. [file 41598_2021_97788_MOESM1_ESM.docx]

Supplementary Table S1. Correlations between emotion valences and clinical characteristics (cognition, disease duration, LEDD) in PD patients.

|  | Negative emotions* | | | Positive emotions | | |
| --- | --- | --- | --- | --- | --- | --- |
|  | *r* | *df* | *p* | *r* | *df* | *p* |
| **Cognition** |  |  |  |  |  |  |
| *Mattis DRS* | -0.30 | 11 | 0.32 | 0.59 | 11 | **0.03** |
| *VOSP (letters)* | -0.29 | 11 | 0.34 | 0.37 | 11 | 0.21 |
| *VOSP (figures)* | -0.42 | 11 | 0.15 | 0.30 | 11 | 0.32 |
| **Disease duration** | 0.28 | 11 | 0.36 | -0.02 | 11 | 0.96 |
| **LEDD** | 0.23 | 7 | 0.56 | 0.20 | 7 | 0.60 |

Pearson correlations coefficients (r), degrees of freedom (df) and p values were indicated in the table.

All p values are > 0.05 after Bonferroni correction.

*correlations between clinical characteristics and emotions valence for each negative emotion (namely anger, sadness and fear) were not significant.

Abbreviations: LEDD: levodopa equivalent daily dose; Mattis DRS: Mattis dementia rating scale. VOSP: Visual Object and Space Perception.
